# Whole genome sequencing of the black grouse (*Tetrao tetrix*): reference guided assembly suggests faster-Z and MHC evolution

**DOI:** 10.1186/1471-2164-15-180

**Published:** 2014-03-06

**Authors:** Biao Wang, Robert Ekblom, Ignas Bunikis, Heli Siitari, Jacob Höglund

**Affiliations:** Department of Ecology and Genetics, Evolutionary Biology Centre, Uppsala University, Norbyvägen 18D, SE-75236 Uppsala, Sweden; Department of Immunology, Genetics and Pathology, Rudbeck Laboratory, Uppsala University, Dag Hammarskjölds väg 20, SE-75237 Uppsala, Sweden; Department of Biological and Environmental Science, University of Jyväskylä, P. O. Box 35, FI-40014 Jyväskylä, Finland

## Abstract

**Background:**

The different regions of a genome do not evolve at the same rate. For example, comparative genomic studies have suggested that the sex chromosomes and the regions harbouring the immune defence genes in the Major Histocompatability Complex (MHC) may evolve faster than other genomic regions. The advent of the next generation sequencing technologies has made it possible to study which genomic regions are evolutionary liable to change and which are static, as well as enabling an increasing number of genome studies of non-model species. However, *de novo* sequencing of the whole genome of an organism remains non-trivial. In this study, we present the draft genome of the black grouse, which was developed using a reference-guided assembly strategy.

**Results:**

We generated 133 Gbp of sequence data from one black grouse individual by the SOLiD platform and used a combination of de novo assembly and chicken reference genome mapping to assemble the reads into 4572 scaffolds with a total length of 1022 Mb. The draft genome well covers the main chicken chromosomes 1 ~ 28 and Z which have a total length of 1001 Mb. The draft genome is fragmented, but has a good coverage of the homologous chicken genes. Especially, 33.0% of the coding regions of the homologous genes have more than 90% proportion of their sequences covered. In addition, we identified ~1 M SNPs from the genome and identified 106 genomic regions which had a high nucleotide divergence between black grouse and chicken or between black grouse and turkey.

**Conclusions:**

Our results support the hypothesis that the chromosome X (Z) evolves faster than the autosomes and our data are consistent with the MHC regions being more liable to change than the genome average. Our study demonstrates how a moderate sequencing effort can be combined with existing genome references to generate a draft genome for a non-model species.

**Electronic supplementary material:**

The online version of this article (doi:10.1186/1471-2164-15-180) contains supplementary material, which is available to authorized users.

## Background

Next generation sequencing (NGS) has spurred a revolution in the development of genomic tools for non-model organisms [1]. In particular, sequencing complete transcriptomes [2] or complexity-reduced fractions of genomes [3] has enabled the identification of genome-wide molecular markers such as single nucleotide polymorphisms (SNPs) and microsatellites (SSRs). Such investigations have also addressed fundamental questions in molecular ecology and evolution, such as the genomic basis for speciation [4, 5], morphological variation [6, 7], disease resistance [8] and selection on life history traits [9, 10].

A complete genome sequence is the ultimate genomic tool for a species. If such a sequence is available it is possible to conduct large-scale, in-depth studies of many important molecular biology processes such as gene expression, transcription regulation, alternative splicing, epigenetic modifications and gene-protein interactions [11–14] which are important in ecological studies. However, applying NGS technologies such as de-novo sequencing on a large eukaryotic genome is still rare, as it represents a considerable investment. The sheer volume of data generated and the computational facilities needed to assemble and analyse it may limit the number of non-specialized labs that are currently able to embark on such a project. However, more whole genome studies are needed to address fundamental questions on the evolution of genome organisation, such as which regions are conserved and which regions change when taxa diverge and become separate species. Published whole NGS genomes of non-model organisms include giant panda [15], cod [16], naked mole rat [17], macaque [18], Tasmanian devil [19], budgerigar [20], Puerto Rican parrot [21], *Heliconius* butterfly [22], Aye-aye [23], collared flycatcher [24], as well as the 29 mammalian genomes recently sequenced at the Broad Institute [25].

The large number of publically available whole genome sequences from both model and non-model organisms can be used to aid genomic investigations in related organisms. One approach is to directly transfer the genomic resources from a model organism to the study species, which would then be called ‘genome enabled taxa’ [26]. This strategy has been used successfully to develop resources such as microsatellite markers [27], SNPs [28], microarrays [29] and exon capture arrays [30]. Alternatively, the genome sequence from a related model organism can be used in the assembly of short read data from the focal species, a process known as reference guided (or reference assisted) assembly [31, 32].

**Figure 1:**
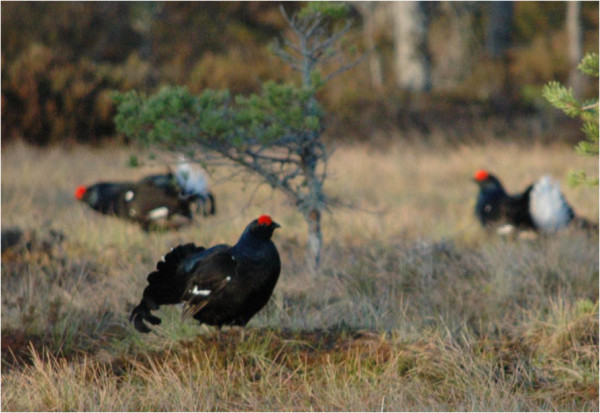
**Male black grouse displaying at a lek.**

Here, we describe a reference guided, whole genome assembly of the black grouse (*Tetrao tetrix*, Figure 1). We take advantage of the close relationship between this species and the well characterized chicken (*Gallus gallus*; both belonging to the order Galliformes with a divergence time of 30 ~ 40 myr) [33–35] and develop a reference guided assembly pipeline to construct a draft genomic sequence. The black grouse is well studied as a model for ecology and conservation biology [36–38] but, until recently, genomic resources for this species have been largely lacking. This work completes our genomic tools development, previously initiated by characterizing the transcriptome [39] and sequencing the major histocompatibility complex (MHC) region [40] in this species.

**Figure 2:**
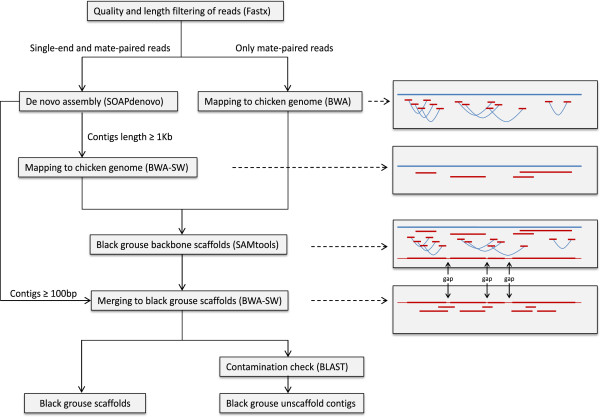
**Flow chart of our reference guided genome assembly pipeline.** All reads were first de-novo assembled. In the second step both long contigs and original mate-paired reads were mapped to the chicken reference genome and merged to produce the black grouse backbone scaffolds. Finally gap-filling was performed by mapping all contigs (of at least 100 bp) back to the backbone scaffolds, producing the draft genome assembly. In addition all contigs (at least 200 bp long) not mapping to the backbone scaffolds were added to the assembly after removing sequences arising from possible contamination using a BLAST approach. For more details about the procedures please see the Methods section. In the figures to the right the chicken reference genome is indicated by the blue line while reads, contigs and scaffolds from the black grouse are shown in red. The light red parts of the final scaffold indicate regions with gaps (Ns) in the black grouse sequence. Within brackets in the boxes to the left are the software used for each different stage of the assembly process.

In theory there are two different ways of using the reference sequence to guide the assembly process. Under an “align-then-assemble” strategy, the reads are first mapped to the reference sequence and clusters of reads mapping to the same location are then extracted and assembled de-novo. Alternatively, in the “assemble-then-align” strategy, the reads are first de-novo assembled and the resulting contigs are then aligned to the reference genome to close gaps and create scaffolds [41]. In our reference guided assembly pipeline (Figure 2) we use a combination of these two approaches by mapping both mate-paired reads and de-novo assembled contigs to the reference genome and combining these alignments to produce the final scaffolds. This approach has some similarities to the previously published “reference-assisted chromosome assembly” [42]. Importantly, we also demonstrate the utility of the SOLiD sequencing technology (Applied Biosystems) for whole genome de-novo sequencing. Due to the short reads produced by this method compared to more widely used 454 (Roche) and HiSeq (Illumina) sequencers, the SOLiD platform has not been used before for sequencing of vertebrate sized genomes in a non-model organism. Bacterial [43] and fungal [44] genomes have, however, previously been sequenced solely based on this technology. Even though the de-novo assembly of our short read data was fragmented (due to the short read lengths) we were still able to successfully cover a large proportion of the genome using our reference guided approach. We used the draft genome both to identify a vast number of SNPs and to perform comparative genomic analyses.

## Results and discussion

### SOLiD sequencing

The raw sequencing data was comprised of 793 M reads with a read length of 75 bp which were generated for the single-end library, 1642 M reads with read length of 60 bp × 60 bp which were generated for the 2 Kb mate-paired library, and 1548 M reads with read length of 60 bp × 60 bp which were generated for the 5 Kb mate-paired library. The raw reads are deposited in the NCBI sequence read archive (SRA) under the accession number SRA061602. After quality and length filtering, 423 M reads (53.3%) were retained for the single-end library, 320 M (75.7%) of which were 75 bp in length. For the 2 Kb mate-paired library, 857 M reads (52.2%) were retained after filtering, and 663 M (77.4%) of them were 60 bp in length. For those filtered reads, 519 M (31.6%) were properly paired, and the rest were only retained as unpaired reads. For the 5 Kb mate-paired library, 847 M reads (54.7%) were retained after filtering, 648 M (76.5%) of which were of 60 bp in length. For those filtered reads, 520 M (33.6%) were properly paired, and the rest were only retained as unpaired reads. Therefore, 2127 M high quality sequencing reads with the total length of approximately 133 Gb were kept in downstream analysis. If we assume that the genome size of black grouse is similar to that of chicken (1.05 G), the estimated mean sequencing coverage of the black grouse genome was 127X.

### Reference guided assembly

The reference guided assembly is comprised of several steps, including *de novo* assembly, reference mapping and the merging of these results (Figure 2). In the first step, all the 2127 M filtered high quality reads were *de novo* assembled by SOAPdenovo. We were able to generate 1298366 preliminary contigs with a total length of 937 Mb. As expected, the *de novo* assembly was more fragmented compared to some other studies which also used short-read sequencing technologies [15, 16, 22], this is because in this study we only had three sequencing libraries with a maximum insert size of 5 Kbp and the sequencing reads produced by the SOLiD technology were relatively short. The SOLiD platform is believed to produce high quality reads [45]. All the filtered data we used in our analyses had an error rate not larger than 0.1%. However, the short read length seriously affects its performance in pure *de novo* assembly. Longer sequencing reads produced by platforms such as 454, ion-torrent or PacBio usually produce larger contigs and such data could be used to improve our assembly in the future.

In the next step, we aligned all long contigs to the chicken genome (Figure 2) and were able to map 277501 of them. The total mapped length was 438 Mb. At the same time, we also aligned the filtered and properly paired reads from the mate-paired libraries to the chicken genome resulting in 451 M successfully mapped reads. These two sets of mapped reads were merged and this resulted in a 805 Mb black grouse genome backbone scaffold. Finally, we mapped the *de novo* assembled contigs back to the black grouse backbone scaffolds and had 1175021 of them mapped. Therefore, we succeeded to cover 833 Mb (79.6%) of the 1046 Mb chicken genome, and 4572 of the 15932 chicken scaffolds (version galGal4). We covered 826 Mb (82.5%) of the 1001 Mb main chicken chromosomes (chromosomes 1-18, and chromosome Z). In addition, we also retained 41098 unmapped contigs (after discarding 265 contigs as likely contaminations) with a total length of 16.6 Mb.

**Figure 3:**
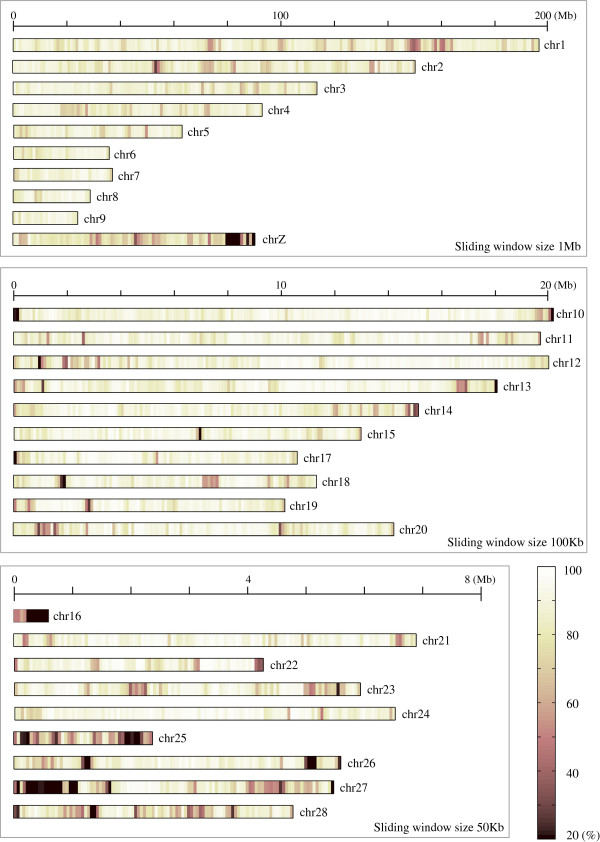
**Heatmap showing the proportion of the regions sequenced on the chromosome scaffolds.**

**Figure 4:**
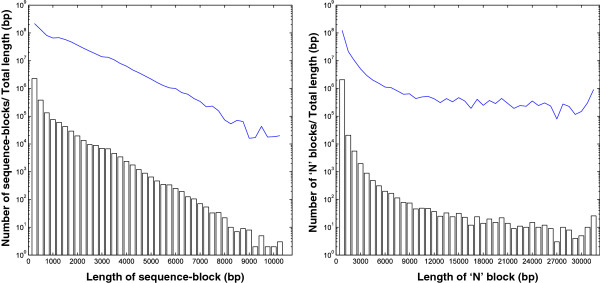
**Distributions of the continuous sequence-blocks and the interleaved ‘N’ blocks.** The blue lines indicate the total length (bp) of the respective blocks. The sequenced blocks larger than 10000 bp and the ‘N’ blocks larger than 30000 bp were binned at the end of the axis.

The resulting black grouse draft genome assembly consisted of 4572 scaffolds with a total length of 1022 Mb (of which 833 Mb is sequenced and the rest represent gaps in the sequence). The genome assembly is deposited in the NCBI whole genome shotgun (WGS) database under the submission number JDSL00000000. Among the scaffolds, the 29 largest, corresponding to the chicken chromosomes 1 ~ 28 and chromosome Z, had a total length of 1001 Mb (826 Mb sequenced). The average coverage (proportion of the sites sequenced) of the 29 chromosomes was 81.5%. However, this coverage was not distributed evenly across the chromosome scaffolds or across the chromosomal regions (Figure 3, Additional file 1). We noticed that chromosome 16, chromosome 25, chromosome 27 and chromosome Z were not well covered. Chromosome Z is the avian sex chromosome and chromosome 16 harbours the MHC genes [35, 46]. These might be more divergent between black grouse and chicken than the rest of the genome, which may have led to the poor assembly. For chromosome 16, an additional reason might be that the chicken assembly of this chromosome is still not perfect and contain large N chunks. Furthermore, we have previously shown that when comparing this region among different galliform species, there are several gene copy divergences as well as genomic inversions in the MHC region on chromosome 16 [40]. We further examined the quality of the black grouse draft genome, and found that although the sequence coverage was high, the scaffold sequences were highly fragmented (Figure 4). If the draft genome is split at all ‘N’ sites present on the scaffolds, it has 3071478 continuous sequenced blocks. This is not unexpected since the SOLiD data was short and we used a reference guided approach to produce the draft genome, and the existing bioinformatic tools available are not mature enough in dealing with this strategy. The SAMtools pipeline we used generates consensus sequences solely based on the coordinates of the reference genome, which might introduce a number of additional ‘N’s in the resulted sequences. In addition, a number of the long ‘N’ chunks are also present in the reference chicken genome, and might thereby be introduced into the black grouse draft genome through the reference guided assembly process.

### Annotation

**Figure 5:**
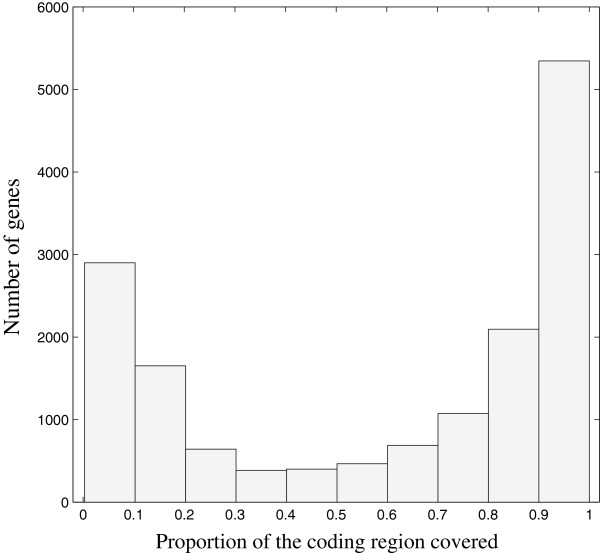
**Histogram of the proportion of the coding regions covered of the annotated black grouse genes.**

The fragmented state of the draft genome limited our ability to systematically perform ab-initio predictions of genes or genomic repeats. Instead we used comparative methods to identify the gene regions and the genomic repetitive regions. From the reciprocal BLAST result, we found that 14826 chicken genes had homologs on the black grouse genome (Table 1, Additional file 2). The coding regions of those homolog genes covered about 45.4 Mb of the black grouse draft genome. We also checked how well each coding sequences of the chicken genes were covered, as this could infer the completeness of the annotated genes of the black grouse genome (Figure 5). We found 5592 genes, with a greater than 90% coverage of the coding regions. This is, however, only a rough estimate, as the length of coding sequences could vary between black grouse genes and chicken genes. We also noticed the interesting ‘U’ shape of the plot, that is, the coding regions of a large majority of the genes are either covered to a very large extent (genes with coverage above 80%) or to a very small extent (genes with coverage less than 20%). This may be explained if some genes that are highly divergent between black grouse and chicken could not be properly aligned in the reference guided assembly step.

**Table 1:** **Number of genes from other bird genomes found to be homologous to the black grouse draft genome**

	Chicken	Turkey	Zebra finch
Number of genes	17934	15006	18618
Black grouse homologs	14826	13721	12573
Percentage (%)	82.7	91.4	67.5

Looking at the distribution of the annotated genes across the scaffolds, we found that the majority of the genes were identified on the 29 main chromosome scaffolds. Interestingly, 634 genes were identified from the unmapped contigs, suggesting that those genes could be not included in the reference chicken genome, or be highly divergent between black grouse and chicken. The average gene density of the 29 main chromosome scaffolds was 1.41E-5 gene/nucleotide. Chromosome 1 had the highest number of genes (2017) as it was the longest chromosome. Chromosome 16 had the highest gene density of 1.03E-4 gene/nucleotide, while chromosome Z had the lowest gene density of 7.99E-6 gene/nucleotide.

Genomic repeats were identified using all known avian genomic repeats as references. We found a total length of 64.7 Mb genomic repeats, which accounted for 6.34% of the draft genome (Table 2). These numbers are lower than those of chicken and zebra finch, but similar to those of turkey [35, 47, 48]. The chicken genome and the zebra finch genome were developed exclusively by Sanger sequencing and have a better quality than that of turkey and black grouse. This could be the reason why we found less genomic repeats in the black grouse draft genome. The black grouse draft genome also had low numbers of simple repeat elements and low complexity regions compared to the other species. However, this can be explained by the fragmented nature of the black grouse draft genome where simple repeats elements and low complexity regions may simply be represented by ‘N’ blocks, preventing the program from detecting them.

**Table 2:** **Information on repeat elements identified from sequenced bird genomes**

	Black grouse	Chicken	Turkey	Zebra finch
Total length of repeats (Mb)	64.7	111.2	60.9	97.5
Percentage in genome (%)	6.34	10.63	5.74	7.91
Number of specific elements				
SINEs	4869	5207	4745	5512
LINEs	151426	183599	140717	133331
L2/CR1/Rex	151298	183464	140575	126284
LTR elements	17792	32080	6325	78514
Retroviral	17733	32015	6266	78185
DNA transposons	19300	21971	3919	4391
Small RNA	1440	1806	1609	1897
Satellites	1433	3816	1247	488
Simple repeats	50145	133371	83093	123377
Low complexity	65102	143762	125682	196866
Unclassified	2017	2222	2028	2334

### Identification of SNPs

Even though the genome sequence is only based on one individual, the fact that this wild, outbred bird was highly heterozygous allowed us to identify a large number of SNPs [15]. To this end, we mapped all the filtered sequencing reads to the black grouse draft genome. 983 M reads, including the ones from the single-end library, the singletons from the mate-paired libraries and the paired reads of the two mate-paired libraries, were mapped to the genome with average coverage of 57.2X. We set the coverage cut-off for the SNP calling as 50 to 100X. We only accepted SNP sites with certain level of coverage to ensure the quality of the SNP calling but also to avoid the sites with unusually high coverage, as they might be the result of incorrect mapping of reads from duplicated regions. Thus, we finally obtained 964054 high quality SNPs, 31993 (3.3%) of which were from the coding regions (Table 3). The transition/transversion ratio of the SNPs was 2.05. The SNP density of this black grouse individual was 0.114%, which was higher than that of turkey (0.064%) [48]. However, the individual used in the turkey genome sequencing project was inbred, whereas the black grouse individual we used was from a large outbred natural population. Interestingly, we found that the SNP density of black grouse was close to that of the giant panda (0.135% for autosomes), which was inferred from a captive but outbred individual [15].

**Table 3:** **Number and density of single nucleotide polymorphisms (SNPs) identified in the genome sequence from one outbred black grouse individual**

	Number of SNP	SNP density (%)	SNP type					
			Transition		Transversion			
			A/G	C/T	A/C	A/T	C/G	G/T
Total	949254	0.114	320842	320314	79442	84824	60193	80439
Macro-chromosomes (1 ~ 5)	601867	0.120	201789	201258	51505	56799	37937	52579
Intermediate-chromosomes (6 ~ 10)	146269	0.117	49447	49574	12547	12736	9554	12411
Micro-chromosomes (11 ~ 28)	161369	0.111	54160	53843	13324	12465	10918	13459
Chromosome Z	39749	0.071	15446	15639	2066	2824	1784	1990

We further investigated the SNPs on the 29 large chromosome scaffolds (Table 3, Additional file 1). We classified the chromosomes into four categories: macro-chromosomes (chromosome 1 ~ 5), intermediate-chromosomes (chromosome 6 ~ 10), micro-chromosomes (chromosome 11 ~ 28) and sex chromosome (chromosome Z). We found that the macro-chromosomes had the highest heterozygosity while the sex chromosome had the lowest. The heterozygosity of the micro-chromosomes was also low. This might be because that the micro-chromosomes have a higher gene density in the black grouse. In contrast, the sex chromosome had the lowest density of genes but also had a low heterozygosity. Similar patterns have been observed in a wide variety of organisms and are explained by the fact that the effective population size of chromosome Z is theoretically 0.75 compared to that of the autosomes [49]. In addition, the reduced variation on the Z (corresponding to X in mammals and flies) could also be interpreted as the result of faster evolution and purifying selection [50–52].

### Comparative genomics

Since the scaffolds of the black grouse draft genome were developed by using the chicken genome as reference, we could not investigate the genomic variations of black grouse, chicken and other species from a genomic rearrangement perspective, however, the sequences allowed us to conduct a comprehensive comparative genomic analysis at the level of nucleotide variation. For this analysis, we focused on the main chromosomes (chromosome 1-28 and chromosome Z) and examined the nucleotide divergence (number of variable sites per unit) between black grouse, chicken and turkey. The downloaded chicken genome was split to 187307 sequences, of which 181105 (96.7%) could be mapped to the black grouse main chromosome scaffolds (chromosome 1-28 and chromosome Z). This alignment covered 795 M (96.2%) of the sequenced sites of the main black grouse chromosomes. The downloaded turkey genome was split to 336344 sequences, of which 328727 (97.7%) could be mapped to the main black grouse chromosome scaffolds. This alignment covered 703 M (85.1%) of the sequenced sites of the main black grouse chromosomes. The turkey genome had a higher mapping percentage but a much lower coverage of the sequenced sites of the black grouse genome, as the turkey genome sequences were of lower quality (containing many unresolved nucleotides ‘N’,) compared to those of chicken.

**Figure 6:**
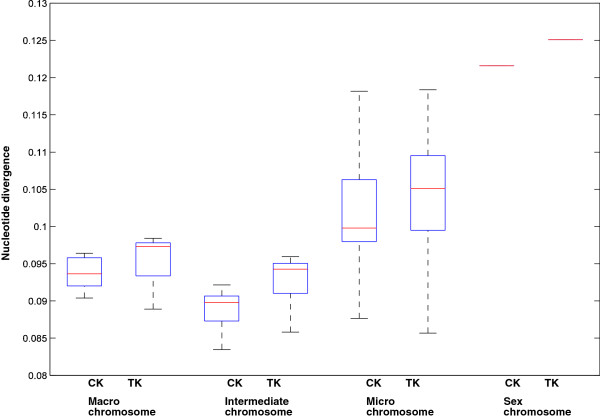
**Nucleotide divergence of the chromosome groups.** Macro-chromosome: chromosome 1 ~ 5. Intermediate-chromosome: chromosome 6 ~ 10. Micro-chromosome: chromosome 11 ~ 28. Sex chromosome: chromosome Z. CK represents the comparison between black grouse and chicken. TK represents the comparison between black grouse and turkey.

The average nucleotide divergence between the 29 black grouse and chicken chromosomes was 0.099 ± 0.009, with the divergence between black grouse and turkey was 0.101 ± 0.009. Those nucleotide divergence estimates were a little lower compared to the studies on chicken and turkey [53, 54], this, however, might be because we used a genome mapping approach to complete the alignment, which could make us miss the most highly divergent sequences. The black grouse, the chicken and the turkey are closely related species. Counter to our findings here, phylogenetic analysis suggests that the black grouse is more closely related to turkey than chicken [33, 46]. This might be because, since we used chicken genome as reference to construct the black grouse draft genome for the heterozygous nucleotide sites, the choice of the nucleotides could be biased towards the chicken reference genome. To further investigate the nucleotide divergence, we grouped the chromosomes into four categories (macro-chromosome, intermediate-chromosome, micro-chromosome, sex chromosome) as described in the last section. We found that the nucleotide divergence of intermediate-chromosomes was slightly lower than that of the macro-chromosomes, and the nucleotide divergence of micro-chromosomes was slightly higher than that of macro-chromosomes and intermediate-chromosomes (Figure 6). The nucleotide divergence of the Z chromosome was also higher than for the autosomes. The observation of increased divergence rates on sex chromosomes is often referred to as the faster X effect [55]. This pattern is generally thought to arise from the smaller effective population size of sex chromosome compared to autosomes, or an increased accumulation of recessive adaptive mutations [50, 52, 56, 57].

**Figure 7:**
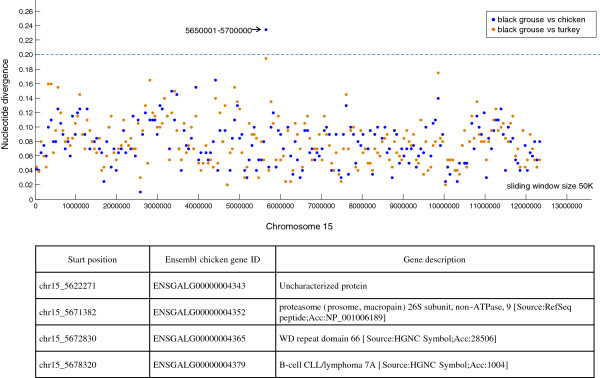
**Example of the identification of the highly divergent regions and the related genes.** The nucleotide divergence was calculated by 50 Kb sliding window. On chromosome 15, the plotted dot ‘5650001-5700000’ of the sliding window was higher than 0.2, so this region was identified as a highly divergent region. Below the genes located on this region were also identified.

Finally, we calculated the nucleotide divergence by 50 Kb sliding window size to screen for the highly divergent genome regions between black grouse and chicken and between black grouse and turkey. A previous study suggested the divergent rate of the galliform MHC region was approximate 0.15 [40]. Here, we used a divergence rate of 0.2 as the cut-off and identified 106 regions which had a high nucleotide divergence rate (exampled in Figure 7, Additional file 3). These regions could potentially harbour genes or gene regulatory elements which are important to some specific phenotypic attributes of the black grouse. Among the identified genomic regions, 45 contained genes or gene fragments and a total of 67 genes were localized in these high divergence regions. Those genes are important to the future in-depth studies of the lineage specific evolution of the black grouse.

## Conclusions

In this study, using the chicken genome as a reference, we successfully assembled the whole draft genome of black grouse. The draft genome consists of 4572 scaffolds with a total length of 1022 Mb (833 Mb sequenced), and additional 41098 unscaffoled contigs with total length of 16.6 Mb. This corresponds to a high coverage of the chicken chromosomes 1 ~ 28 and chromosome Z, with a total length of 1001 Mb (826 Mb sequenced). Although the continuously sequenced blocks on the scaffolds are fragmented, the draft genome has a good coverage of the homologous chicken genes, and 14826 (82.7%) of the chicken genes were identified on the black grouse draft genome. Notably, 33.0% of the coding regions of the homologous genes have more than 90% proportion of their sequences covered. To our knowledge, this is the first time a large eukaryote genome was developed by SOLiD short sequencing technology and reference guided assembly bioinformatic pipeline. Our study demonstrates how a moderate sequencing effort can be combined with existing genome references to accomplish a large genome project. We identified a large number (949254) of SNPs and identified the genomic regions we suggest are important for the lineage specific evolution of black grouse. From the above analysis, we note that the sex chromosome (chromosome Z) had lower reference assembly efficiency, lower SNP density but a higher nucleotide divergence between black grouse and other galliform species. Those multiple evidences support the faster X (Z) hypothesis of the sex chromosome, which states that the chromosome X (Z) evolves faster than the autosomes due to its lower effective population size and recombination rate. We also observed that microchromosome 16 which harbours the MHC region in galliforms was highly divergent among species which may indicate faster evolution in this genomic region.

## Methods

### DNA sampling, extraction and sequencing

The black grouse individual used in this study was a male collected by a licensed hunter in the winter hunting season of 2011 in Hundhamaren, Norway, where a large and continuously distributed black grouse population resides. The fresh blood of the sample was immediately stored in RNAlater (Ambion). DNA extraction was performed using DNeasy Blood & Tissue Kit (Qiagen) following the manufacturer’s instructions. The library preparations and genome sequencing was performed at the Uppsala Genome Centre (http://www.igp.uu.se/facilities/genome_center/) using the Applied Biosystems SOLiD 5500xl platform. One single-end library with a read length of 75 bp, one mate-paired library with an insertion size of 2 Kb and read length of 60 × 60 bp, and one mate-paired library with an insertion size of 5 Kb and read length of 60 × 60 bp were constructed. Each library was sequenced on a full flowchip which contained six lanes. Both versions (colour-space/base-space) of the sequencing reads were obtained from the sequencing centre.

### Preliminary *de novo*assembly

To make the best use of existing NGS analysis tools, we employed the widely used base-space version of data in all our bioinformatic analysis. The raw reads were first quality and size filtered using FASTX-Toolkit (http://hannonlab.cshl.edu/fastx_toolkit/). The threshold of the FASTQ quality score was set at 30; the thresholds of the length of the trimmed reads were 60 bp for the single-end library and 50 bp for the two mate-paired libraries. The filtered mate-paired reads were paired again using a custom made script.

All the sequencing reads were initially *de novo* assembled using SOAPdenovo v 1.05 (63mer version) [58] with default settings (Additional file 4). The assembly was performed on Uppmax Halvan cluster with 64 parallel threads and 2048 GB memory (http://www.uppmax.uu.se/halvan). We tested K-mer size exhaustively from 15 to 55, stepped by 2, and accepted the result with the longest N50 for the downstream mapping analysis. K-mer 31 gave the best result in this regard. Using it, we generated 1298366 preliminary contigs with length not shorter than 100 bp. The longest contig was 12574 bp in length. The average length of the contigs was 722 bp, and the contig N50 size was 1238 bp. The depths of coverage of the preliminary contigs ranged from 10 to 153, with the average of 35.1. The de-novo assembly scaffolds had an average length of 6010 bp. The longest was 53114 bp, and the N50 size of was 2065 bp.

### Reference guided assembly and mapping

In order to improve the preliminary assembly we developed a reference guided approach (Figure 2). The well-established chicken genome (ICGSC Gallus_gallus-4.0/galGal4) [35], which was downloaded from the UCSC genome browser database [59], was used as the reference genome. To avoid incorrect mapping of the short sequences onto rearranged genome regions between black grouse and chicken, only preliminary contigs of 1 Kb or larger (335884 contigs with a mean length of 1817 bp) were selectively mapped. The mapping was performed using BWA-SW algorithm [60, 61] implemented in the Burrows-Wheeler Aligner (BWA) package v0.6.2. The BWA-SW algorithm was designed to enable the alignment of long sequences (up to 1 Mb) against a large sequence database at a relatively fast speed. To customize the algorithm to our needs we decreased the Gap extension penalty score (-r) to 1, as long trunks of insertions and deletions had been observed between the sequences of black grouse and chicken [46].

In parallel, we mapped the filtered and properly paired sequencing reads from the 2 Kb mate-paired library and the 5 Kb mate-paired library onto the reference chicken genome. We only adopted the mate-paired libraries because we wanted, as much as possible, to avoid incorrect mapping caused by genomic rearrangements between black grouse and chicken. The Burrows-Wheeler Aligner (BWA) [62] program v0.6.2 was used to conduct the mapping and custom alignment settings of Maximum edit distance (-n) 5, Maximum number of gap opens (-o) 2, Maximum number of gap extensions (-e) 10, Gap open penalty (-O) 8, and Gap extension penalty (-E) 2 were configured to make the program more tolerant to the indel variation between black grouse and chicken [39, 40]. The alignments were then summarized using the ‘bwa sampe’ command. The program automatically estimated the insertion size and direction between the paired reads and discarded the inferred incorrectly mapping pairs. The coverage of the alignment was estimated and the over-low/high covered sites were discarded by a custom made script to avoid incorrect mapping introduced by random factors or piling up of reads from duplicated genomic regions.

### Reference guided assembly, merging and finalising

The BAM format alignment files of the contig mapping and the mate-pair read mapping were subsequently merged using SAMtools suite v0.1.18 [63]. Then, the consensus sequences of black grouse were extracted from the merged alignment file by the ‘samtools mpileup’, ‘bcftools’ and ‘vcfutils.pl’ (vcf2fq) pipelines from the SAMtools suite. We used the consensus sequences of the black grouse scaffolds as a backbone to map all the contigs (not shorter than 100 bp) generated from the *de novo* assembly in order to further close gaps in the scaffolds and extend the sequenced regions (non-N) of the draft genome. The mapping was performed using BWA-SW program with its default configuration. To make use of the SAMtools consensus generating pipeline, the backbone scaffolds were split into 10 Kb fragments and mapped back onto themselves also using the BWA-SW program. The resulting alignment was merged with the contigs mapping alignment using SAMtools. This merged alignment was used to generate the final black grouse draft genome using the SAMtools pipeline. The remaining 41363 unmapped contigs (not smaller than 200 bp) were extracted and aligned to the NCBI Nucleotide collection (nt) and Genome survey sequence (gss) databases using BLASTN of the NCBI BLAST 2.2.27+ package [64]. We discarded sequences of non-avian origin according to the BLAST search as they might be contamination. The remaining contig sequences were kept separately as parts of the black grouse draft genome.

### Annotation

The annotation of genes and genomic repeats was conducted by comparative methods. To identify genes, we downloaded the chicken genes (WASHUC2) from the Ensembl database [65] and followed a reciprocal BLAST approach to align the chicken genes and the black grouse draft genome. We firstly aligned the chicken cDNA sequences to the black grouse genome using the BLASTN program from the NCBI BLAST 2.2.27+ package. The E-value cut-off was set as 10E-10. We then extracted the aligned sequences from the black grouse genome and aligned them to the chicken proteins using the BLASTX program. The BLAST results were compared using a self-written script to keep only the reciprocal BLAST hits. Using the same BLAST protocol, we also searched the homologous turkey and zebra finch genes along the black grouse draft genome. The entire sets of the turkey proteins (UMD2) [48] and the zebra finch proteins (taeGut3.2.4) [47] were also downloaded from the Ensembl database. Since the chicken genome was released earliest and has the most direct molecular biology support for the genes [35], we accepted the BLAST result of chicken as the annotation of the black grouse genes.

To identify genomic repeats, we used the RepeatMasker program (http://www.repeatmasker.org/) to scan the black grouse draft genome sequence. RMBlast (RepearMasker compatible version of NCBI BLAST) (http://www.repeatmasker.org/RMBlast.html) was used as the alignment engine. The RepeatMasker library v20120418 was downloaded from RepBase (http://www.girinst.org/server/RepBase/index.php) and we specified the species library as ‘aves’ for the black grouse. For a comparative purpose, we also ran the RepeatMasker analysis for the latest versions of the chicken genome (galGal4), the turkey genome (melGal1) and the zebra finch genome (taeGut1), which were downloaded from the UCSC genome browser database.

### Identification of SNPs

To identify SNPs present as heterozygous sites in our one outbred male black grouse, we first mapped all the filtered reads, including those from the single-end library, the paired reads and the singletons from the two mate-paired libraries to the black grouse draft genome using BWA v0.6.2. The alignment was performed using the ‘bwa aln’ command with default settings, ‘bwa samse’ with default settings was subsequently used for the reads of the single-end library and the singletons from the mate-paired libraries, and ‘bwa sampe’ with default settings was used for the paired reads of the two mate-paired libraries. The alignment files generated from the mapping were then merged together using SAMtools utilities v0.1.18. The average depth of coverage of the mapped sites was estimated from the SAM file and was used to determine the coverage cut-off of the SNP calling. The SNP calling followed the ‘samtools mpileup’, ‘bcftools’ and ‘vcfutils.pl’ (varFilter) pipelines. The Bayesian inference of the variants (-b) was enabled in ‘bcftools’. The statistics of the identified SNPs was calculated and evaluated using custom made scripts.

### Comparative genomics

For the comparative genomic analysis at the level of nucleotide divergence, we focused on the chromosome scaffolds (chromosome 1-28 and chromosome Z). The chromosome sequences of chicken (galGal4) and turkey (melGal1) were downloaded from USCS genome browser database. Since directly aligning large genomic sequences is a cumbersome and time-consuming task, we split the genomic sequences of chicken and turkey into 10 Kb pieces, and then aligned these short sequences to the black grouse draft genome (chromosome 1-28 and chromosome Z) using the BWA-SW program with settings of Gap open penalty (-q) 1 and Gap extension penalty (-r) 1. The sequences with alignment depth of coverage more than 1 were excluded in downstream analysis. All the nucleotide variants were summarized using ‘SAMtools mpileup’ and ‘bcftools’ pipelines with probabilistic realignment for the computation of base alignment quality (BAQ) disable (-B). The statistics of the nucleotide divergence (percentage of variable sites per sequence) was calculated from the Variant call format (VCF) file by custom made scripts.We also used a sliding window (50 Kb) approach to scan the highly divergent regions across the genomes between black grouse/chicken, black grouse/turkey to identify the genomic regions which might be important in the lineage specific evolution of black grouse.

### Data accessibility

Raw sequencing reads: NCBI sequence read archive (SRA) SRA061602 Genome assembly: NCBI whole genome shotgun (WGS) database JDSL00000000

## Electronic supplementary material

Additional file 1: **The details of assembly, gene annotation, SNP discovery and nucleotide divergence of chromosome 1 ~ 28, and chromosome Z.** (PDF 107 KB)

Additional file 2: **The annotation list of the black grouse genes.** (TXT 576 KB)

Additional file 3: **The highly divergent genomic regions with related genes identified by the 50 Kb sliding window.** (PDF 250 KB)

Additional file 4: **Comparison of the first-step assembly using different programs.** (PDF 184 KB)
