# Supplementary material for: Whole genome sequencing of the black grouse (Tetrao tetrix): reference guided assembly suggests faster-Z and MHC evolution
Source: BMC Genomics. 2014 Mar 6;15(1):180. doi: 10.1186/1471-2164-15-180 (PMC4022176; doi:10.1186/1471-2164-15-180)
Supplement: Supplementary file 1 — Additional file 1: The details of assembly, gene annotation, SNP discovery and nucleotide divergence of chromosome 1 ~ 28, and chromosome Z. (PDF 107 KB) [file 12864_2013_7022_MOESM1_ESM.pdf]

Additional file 1. The details of assembly, gene annotation, SNP discovery and nucleotide divergence of chromosome 1~28, and chromosome Z.

chromosome	scaffold length	sequenced length	sequenced percentage	number of sequence-block	average length of sequence-block	number of N block	average length of N block	number of genes	gene desity (×10E-6)	number of SNPs	SNP density (%)	average nucleotide divergence between black grosue and chicken	average nucleotide divergence between black grosue and turkey
chr1	195244616	158728977	0.813	622352	255.0	622351	58.7	2365	12.1	197846	0.125	0.096	0.097
chr2	148806577	121771735	0.818	450947	270.0	450946	60.0	1455	9.8	146231	0.120	0.094	0.095
chr3	110440357	94045792	0.852	338535	277.8	338534	48.4	1292	11.7	112889	0.120	0.096	0.098
chr4	90169680	75127893	0.833	281184	267.2	281183	53.5	1143	12.7	84743	0.113	0.090	0.089
chr5	59549754	50581140	0.849	180834	279.7	180833	49.6	955	16.0	60158	0.119	0.093	0.098
chr6	34906596	30004276	0.860	103486	289.9	103485	47.4	554	15.9	35496	0.118	0.090	0.096
chr7	36213568	31594475	0.872	108025	292.5	108024	42.8	522	14.4	36918	0.117	0.090	0.095
chr8	28700717	25060471	0.873	82230	304.8	82229	44.3	566	19.7	28450	0.114	0.092	0.094
chr9	23430632	20643053	0.881	69264	298.0	69263	40.2	449	19.2	24326	0.118	0.089	0.093
chr10	19907917	17581023	0.883	56919	308.9	56918	40.9	525	26.4	21079	0.120	0.083	0.086
chr11	19396707	17015365	0.877	54903	309.9	54902	43.4	422	21.8	19087	0.112	0.099	0.107
chr12	19861678	17341655	0.873	57729	300.4	57728	43.7	352	17.7	20958	0.121	0.093	0.102
chr13	17757989	15343371	0.864	53575	286.4	53574	45.1	378	21.3	17540	0.114	0.101	0.110
chr14	15116539	12974155	0.858	46871	276.8	46870	45.7	455	30.1	14248	0.110	0.109	0.104

chr15	12642258	11163622	0.883	36509	305.8	36508	40.5	404	32.0	13173	0.118	0.100	0.104
chr16	530826	117833	0.222	1110	106.2	1109	372.4	71	133.8	32	0.027	0.088	0.092
chr17	10441543	9117519	0.873	29990	304.0	29989	44.2	333	31.9	9480	0.104	0.099	0.106
chr18	11199738	9408799	0.840	32899	286.0	32898	54.4	306	27.3	10221	0.109	0.105	0.090
chr19	9982233	8639582	0.865	29121	296.7	29120	46.1	320	32.1	9631	0.111	0.099	0.102
chr20	14272843	12123883	0.849	40810	297.1	40809	52.7	354	24.8	13749	0.113	0.118	0.112
chr21	6797072	5989529	0.881	21253	281.8	21252	38.0	256	37.7	6469	0.108	0.111	0.114
chr22	4076697	3497232	0.858	13228	264.4	13227	43.8	129	31.6	3994	0.114	0.093	0.093
chr23	5718086	4712397	0.824	18788	250.8	18787	53.5	241	42.1	4273	0.091	0.096	0.086
chr24	6320252	5666250	0.897	19166	295.6	19165	34.1	204	32.3	5286	0.093	0.118	0.107
chr25	2189425	1149133	0.525	7501	153.2	7500	138.7	139	63.5	946	0.082	0.099	0.118
chr26	5328732	4308818	0.809	18073	238.4	18072	56.4	242	45.4	4502	0.104	0.105	0.105
chr27	5202594	3409551	0.655	17125	199.1	17124	104.7	245	47.1	3982	0.117	0.103	0.109
chr28	4735135	3467941	0.732	15542	223.1	15541	81.5	220	46.5	3798	0.110	0.102	0.104
chrZ	82360923	55779024	0.677	263509	211.7	263508	100.9	709	8.6	39749	0.071	0.122	0.125
All	1021612514	832752756	0.815	3071478	266.6	3071449	60.5	15606	15.3	949254	0.114	0.099	0.101
