# Supplementary material for: Whole genome sequencing of the black grouse (Tetrao tetrix): reference guided assembly suggests faster-Z and MHC evolution
Source: BMC Genomics. 2014 Mar 6;15(1):180. doi: 10.1186/1471-2164-15-180 (PMC4022176; doi:10.1186/1471-2164-15-180)
Supplement: Supplementary file 3 — Additional file 3: The highly divergent genomic regions with related genes identified by the 50 Kb sliding window. (PDF 250 KB) [file 12864_2013_7022_MOESM3_ESM.pdf]

Additional file 3. The highly divergent genomic regions with related genes identified by the 50Kb sliding window.

Chromosome and start position	Nucleotide divergence BG vs CK	Nucleotide divergence BG vs TK	Ensembl gene ID	Gene description
chr1				
6700000	0.196	0.212	ENSGALG00000006656	FERM domain containing 4A [Source:HGNC Symbol;Acc:25491]
12750000	0.200	0.208	ENSGALG00000008249	DnaJ (Hsp40) homolog, subfamily C, member 2 [Source:HGNC Symbol;Acc:13192]
			ENSGALG00000008236	proteasome (prosome, macropain) 26S subunit, ATPase, 2 [Source:RefSeq peptide;Acc:NP_001006225]
			ENSGALG00000008227	Uncharacterized protein [Source:UniProtKB/TrEMBL;Acc:F1NDR8]
			ENSGALG00000008222	Uncharacterized protein [Source:UniProtKB/TrEMBL;Acc:F1NDS6]
14300000	0.196	0.210	ENSGALG00000008050	HMG-box transcription factor 1 [Source:HGNC Symbol;Acc:23200]
			ENSGALG00000008035	component of oligomeric golgi complex 5 [Source:HGNC Symbol;Acc:14857]
24050000	0.194	0.210	ENSGALG00000009104	Uncharacterized protein [Source:UniProtKB/TrEMBL;Acc:E1BZ82]
			ENSGALG00000009110	N(alpha)-acetyltransferase 38, NatC auxiliary subunit [Source:HGNC Symbol;Acc:20471]
57000000	0.200	0.182	ENSGALG00000012863	aarF domain containing kinase 2 [Source:HGNC Symbol;Acc:19039]
			ENSGALG00000023078	NADH dehydrogenase (ubiquinone) 1 beta subcomplex, 2, 8kDa [Source:HGNC Symbol;Acc:7697]
			ENSGALG00000012864	Uncharacterized protein [Source:UniProtKB/TrEMBL;Acc:F1P1M4]
70050000	0.204	0.194	ENSGALG00000014218	PHD finger protein 21B [Source:HGNC Symbol;Acc:25161]
74900000	0.178	0.204	ENSGALG00000014346	tetraspanin 9 [Source:HGNC Symbol;Acc:21640]
77450000	0.208	0.208	ENSGALG00000014749	EPH receptor B6 [Source:RefSeq peptide;Acc:NP_001004387]
104550000	0.198	0.200	ENSGALG00000015938	interferon (alpha, beta and omega) receptor 2 [Source:RefSeq peptide;Acc:NP_990189]
104700000	0.196	0.200	ENSGALG00000015983	phosphoribosylglycinamide formyltransferase, phosphoribosylglycinamide synthetase, phosphoribosylaminoimidazole synthetase [Source:RefSeq peptide;Acc:NP_001001469]
104800000	0.172	0.200	ENSGALG00000015992	crystallin, zeta (quinone reductase)-like 1 [Source:HGNC Symbol;Acc:2420]
129450000	0.182	0.204	ENSGALG00000016727	PHD finger protein 16 [Source:HGNC Symbol;Acc:22982]
chr2				
25050000	0.206	0.178	ENSGALG00000010704	Uncharacterized protein [Source:UniProtKB/TrEMBL;Acc:F1NS19]
27400000	0.188	0.204	ENSGALG00000010780	ets variant gene 1 [Source:RefSeq peptide;Acc:NP_990248]
95500000	0.210	0.206	ENSGALG00000013782	cadherin 7, type 2 [Source:RefSeq peptide;Acc:NP_989518]

97100000	0.220	0.220	ENSGALG00000013853	Uncharacterized protein [Source:UniProtKB/TrEMBL;Acc:E1BZ74]
102800000	0.194	0.200	ENSGALG00000015056	laminin, alpha 3 [Source:HGNC Symbol;Acc:6483]
chr3				
22200000	0.168	0.208	ENSGALG00000009896	transcriptional regulating factor 1 [Source:HGNC Symbol;Acc:18273]
44850000	0.212	0.182	ENSGALG00000011624	poly(A)-specific ribonuclease (PARN)-like domain containing 1 [Source:HGNC Symbol;Acc:21185]
			ENSGALG00000011625	mitochondrial ribosomal protein L18 [Source:HGNC Symbol;Acc:14477]
			ENSGALG00000017853	Small nucleolar RNA SNORA29 [Source:RFAM;Acc:RF00429]
			ENSGALG00000011637	t-complex 1 [Source:RefSeq peptide;Acc:NP_001006405]
			ENSGALG00000025629	Small nucleolar RNA SNORA20 [Source:RFAM;Acc:RF00401]
			ENSGALG00000011659	acetyl-Coenzyme A acetyltransferase 2 (acetoacetyl Coenzyme A thiolase) [Source:RefSeq peptide;Acc:NP_001034376]
44950000	0.248	0.244	ENSGALG00000011660	Wilms tumor 1 associated protein [Source:HGNC Symbol;Acc:16846]
			ENSGALG00000011661	superoxide dismutase 2, mitochondrial [Source:RefSeq peptide;Acc:NP_989542]
47900000	0.202	0.222	ENSGALG00000012395	pleckstrin homology domain containing, family G (with RhoGef domain) member 1 [Source:HGNC Symbol;Acc:20884]
50350000	0.204	0.192	ENSGALG00000023054	Uncharacterized protein [Source:UniProtKB/TrEMBL;Acc:F1NKH8]
74500000	0.184	0.200	ENSGALG00000015596	Uncharacterized protein [Source:UniProtKB/TrEMBL;Acc:E1C063]
chr4				
17650000	0.188	0.202	ENSGALG00000009096	myotubularin 1 [Source:HGNC Symbol;Acc:7448]
19050000	0.196	0.210	ENSGALG00000009192	SLIT and NTRK-like family, member 2 [Source:HGNC Symbol;Acc:13449]
19150000	0.214	0.192	ENSGALG00000009239	Uncharacterized protein [Source:UniProtKB/TrEMBL;Acc:F1NUH0]
			ENSGALG00000009237	Toll-like receptor 2 type-1 [Source:UniProtKB/Swiss-Prot;Acc:Q9DD78]
23550000	0.220	0.192	ENSGALG00000009567	tolloid-like 1 [Source:RefSeq peptide;Acc:NP_990034]
54100000	0.180	0.208	ENSGALG00000011990	ubiquitin specific peptidase 53 [Source:HGNC Symbol;Acc:29255]
			ENSGALG00000020171	Uncharacterized protein [Source:UniProtKB/TrEMBL;Acc:E1C6U0]
chr5				
14500000	0.206	0.208	ENSGALG00000006799	adaptor-related protein complex 2, alpha 2 subunit [Source:RefSeq peptide;Acc:NP_001012914]
			ENSGALG00000006830	Uncharacterized protein [Source:UniProtKB/TrEMBL;Acc:F1NER5]
22550000	0.198	0.202	ENSGALG00000008255	chromosome 11 open reading frame 49 [Source:HGNC Symbol;Acc:28720]
35350000	0.240	0.244	ENSGALG00000023135	signal recognition particle 54kDa [Source:HGNC Symbol;Acc:11301]
			ENSGALG00000010046	hypothetical protein LOC423323 [Source:RefSeq peptide;Acc:NP_001026373]
			ENSGALG00000010052	protein phosphatase 2, regulatory subunit B", gamma [Source:HGNC Symbol;Acc:17485]
35450000	0.232	0.240	ENSGALG00000010063	Rel-associated pp40 [Source:RefSeq peptide;Acc:NP_001001472]

35600000	0.240	0.238	ENSGALG00000010093	breast cancer metastasis-suppressor 1-like [Source:RefSeq peptide;Acc:NP_001007936]
chr6				
4250000	0.180	0.208	ENSGALG00000002543	chondroitin sulfate N-acetylgalactosaminyltransferase 2 [Source:HGNC Symbol;Acc:24292]
4300000	0.200	0.216	ENSGALG00000002555	ret proto-oncogene [Source:RefSeq peptide;Acc:NP_990521]
6900000	0.212	0.200		
chr8				
50000	0.194	0.202	ENSGALG00000021244	Uncharacterized protein [Source:UniProtKB/TrEMBL;Acc:E1BYX1]
2500000	0.188	0.202	ENSGALG00000002305	zinc finger and BTB domain containing 41 [Source:HGNC Symbol;Acc:24819]
7700000	0.208	0.204	ENSGALG00000004775	glycosyltransferase 25 domain containing 2 [Source:HGNC Symbol;Acc:16790]
			ENSGALG00000004782	tRNA splicing endonuclease 15 homolog (S. cerevisiae) [Source:HGNC Symbol;Acc:16791]
			ENSGALG00000004784	Uncharacterized protein [Source:UniProtKB/TrEMBL;Acc:F1NG28]
chr11				
9850000	0.174	0.204	ENSGALG00000004899	Uncharacterized protein [Source:UniProtKB/TrEMBL;Acc:F1NIQ9]
chr15				
5650000	0.204	0.188	ENSGALG00000004343	Uncharacterized protein [Source:UniProtKB/TrEMBL;Acc:F1NVJ0]
			ENSGALG00000004352	proteasome (prosome, macropain) 26S subunit, non-ATPase, 9 [Source:RefSeq peptide;Acc:NP_001006189]
			ENSGALG00000004365	WD repeat domain 66 [Source:HGNC Symbol;Acc:28506]
			ENSGALG00000004379	B-cell CLL/lymphoma 7A [Source:HGNC Symbol;Acc:1004]
			ENSGALG00000025437	gga-mir-762 [Source:miRBase;Acc:MI0008209]
chr18				
5600000	0.256	0.276	ENSGALG00000003007	Uncharacterized protein [Source:UniProtKB/TrEMBL;Acc:F1NAQ6]
chr20				
700000	0.268	0.240	ENSGALG00000001420	hypothetical protein LOC419128 [Source:RefSeq peptide;Acc:NP_001026017]
chrZ				
1500000	0.198	0.210	ENSGALG00000010056	Uncharacterized protein [Source:UniProtKB/TrEMBL;Acc:F1N881]
15000000	0.202	0.204	ENSGALG00000014884	Uncharacterized protein [Source:UniProtKB/TrEMBL;Acc:E1BWH2]
