# Supplementary material for: Whole genome sequencing of the black grouse (Tetrao tetrix): reference guided assembly suggests faster-Z and MHC evolution
Source: BMC Genomics. 2014 Mar 6;15(1):180. doi: 10.1186/1471-2164-15-180 (PMC4022176; doi:10.1186/1471-2164-15-180)
Supplement: Supplementary file 4 — Additional file 4: Comparison of the first-step assembly using different programs. (PDF 184 KB) [file 12864_2013_7022_MOESM4_ESM.pdf]

Additional file 4 – Comparison of the first-step assembly using different programs.

	Velvet	ABySS	SOAPdenovo
**Number of contigs**	987378	1178635	1298366
**Total length (bp)**	847973941	897476852	937254489
**Average length (bp)**	709	657	722
**N50 (bp)**	1081	933	1238
